# Resveratrol in Oral Squamous Cell Carcinoma: Preclinical Evidence and Translational Opportunities

**DOI:** 10.32604/or.2026.079642

**Published:** 2026-06-16

**Authors:** Alessandro Polizzi, Gaetano Isola, Monia Cecati, Nicoletta Bonci, Roberto Campagna, Giovanni Tossetta

**Affiliations:** 1 Department of General Surgery and Surgical-Medical Specialties, School of Dentistry, University of Catania, Catania, Italy; 2 Department of Human Sciences and Promotion of the Quality of Life, San Raffaele Roma University, Rome, Italy; 3 Department of Experimental and Clinical Medicine, Polytechnic University of Marche, Ancona, Italy

**Keywords:** Resveratrol, stilbenes, polydatin, pinostilbene hydrate, oral squamous cell carcinoma

## Abstract

Polyphenolic stilbenes are plant-derived compounds that have attracted increasing interest for their potential anticancer properties. Among them, resveratrol is the most extensively investigated molecule. Oral squamous cell carcinoma (OSCC) represents a major global health challenge due to its aggressive biological behavior, frequent late diagnosis, and limited improvement in survival outcomes despite advances in treatment. This review aims to summarize current experimental evidence on the anticancer effects of resveratrol in OSCC, also considering structurally related derivatives such as polydatin and pinostilbene hydrate. A structured review of the literature was performed to identify experimental studies investigating the activity of these compounds in OSCC models. Overall, the available evidence indicates that resveratrol exerts multiple antitumor effects in OSCC, including inhibition of cell proliferation, induction of apoptosis and ferroptosis, cell-cycle arrest, and suppression of migration, invasion, and epithelial–mesenchymal transition. These effects are associated with the modulation of several oncogenic signaling pathways and components of the tumor microenvironment. In addition, resveratrol has been reported to enhance the efficacy of chemotherapy, radiotherapy, and targeted therapies, and to counteract mechanisms of drug resistance. However, most findings derive from *in vitro* studies using concentrations that may not be readily achievable *in vivo* due to limited bioavailability. Further *in vivo* and clinical investigations are therefore required to clarify the translational potential of resveratrol in OSCC management.

## 1 Introduction

Stilbenoids represent a diverse and biologically active class of naturally occurring polyphenolic compounds characterized by a 1,2-diphenylethylene backbone and various hydroxylation patterns that confer distinctive chemical properties [1,2]. They are produced primarily by plants as phytoalexins which are defensive metabolites synthesized in response to stress stimuli such as pathogen invasion, UV radiation, or physical injury [3,4]. This inducible nature contributes to the considerable structural variability found within the group, which includes monomeric stilbenes like resveratrol as well as a wide range of oligomeric and polymeric derivatives such as viniferins, pallidol, and hopeaphenol [5]. Their molecular diversity is closely tied to biosynthetic pathways involving stilbene synthase, a key enzyme competing with chalcone synthase within the phenylpropanoid pathway. Small modifications in enzymatic conditions or stress signals can dramatically shift the balance of metabolites produced, making stilbenoids a chemically flexible family that reflects the adaptive strategies of their plant origins [6].

Chemically, stilbenoids are distinguished by their conjugated structure, which supports antioxidant activity and facilitates interactions with a range of cellular targets [1]. The presence, number, and position of hydroxyl groups influence their solubility, redox potential, and overall bioactivity. For example, the *trans* configuration of stilbenes typically confers greater stability and biological potency than their *cis* counterparts. Many stilbenoids also undergo glycosylation, methylation, or oligomerization, transformations that can enhance their stability or bioavailability in living systems. These structural differences directly affect the pharmacological behavior of stilbenoids, including their absorption, metabolism, and capacity to modulate signaling pathways relevant to human disease [7,8].

Interest in stilbenoids has grown significantly in biomedical research due to their broad spectrum of biological activities, particularly their potential anticancer effects. Early attention was drawn to their antioxidant and anti-inflammatory properties, but subsequent studies have demonstrated that stilbenoids can influence key processes underlying carcinogenesis and tumor progression [9]. They have been shown to modulate oxidative stress responses, inhibit pro-inflammatory mediators, and interfere with the activation of oncogenic transcription factors [10]. In many cancer models, stilbenoids can induce cell cycle arrest, promote apoptosis through intrinsic and extrinsic pathways, and disrupt angiogenesis and metastasis [11]. Their ability to interact with multiple molecular targets makes them attractive candidates for prevention strategies as well as for combination therapies aimed at overcoming resistance to conventional agents [9].

In particular, the capacity of stilbenoids to affect redox-sensitive signaling cascades and regulatory proteins such as nuclear factor kappa-light-chain-enhancer of activated B cells (NF-κB), signal transducer and activator of transcription 3 (STAT3), phosphoinositide 3-kinase/protein kinase B (PI3K/AKT), and mitogen-activated protein kinases (MAPKs) has drawn strong interest in oncology [12,13,14]. Many of these pathways play pivotal roles in maintaining malignant phenotypes, supporting survival under stress, and enabling invasive behavior. Stilbenoids often act pleiotropically, counteracting these pathways at several levels without the high toxicity typically associated with synthetic chemotherapeutics. Furthermore, their natural origin and presence in dietary sources have boosted interest in their use also as chemopreventive agents. Although bioavailability remains a challenge for some molecules in this class, ongoing efforts involving nanoparticle delivery, structural modification, and prodrug development continue to expand their therapeutic potential [10].

Collectively, stilbenoids stand out as a promising group of phytochemicals with multifaceted biological activities. Their structural diversity, adaptive biosynthesis, and broad mechanistic repertoire have positioned them as valuable candidates for anticancer research [9]. As investigations evolve from basic mechanistic studies toward more targeted applications, stilbenoids continue to attract attention as potential modulators of tumor behavior and as templates for designing novel therapeutic agents. Oral squamous cell carcinoma (OSCC) represents a particularly suitable setting for translation of such agents because oral lesions are accessible for topical administration and repeated tissue sampling, enabling pharmacodynamic monitoring and biomarker-driven patient stratification in early-phase trials. Among the many stilbenoids investigated, resveratrol has emerged as the most promising molecule, and its anticancer properties have been extensively studied in numerous malignancies. In this review, we will focus on the available literature regarding the studies that investigated the anticancer properties of resveratrol for the management of OSCC. The aim of this review is to summarize and critically discuss the current evidence on the molecular mechanisms and therapeutic potential of resveratrol in OSCC.

## 2 Methodology

To identify studies evaluating the effects of resveratrol and its derivatives in OSCC, a structured literature search was conducted in the PubMed database (https://pubmed.ncbi.nlm.nih.gov/) up to January 2026. The search strategy combined keywords and Boolean operators as follows: ((resveratrol OR polydatin OR stilben OR stilbenoid) AND (“oral squamous cell carcinoma” OR OSCC OR “oral cancer”)).

Only articles published in English were included, and no restrictions on publication year were applied. All retrieved records were exported for screening, and duplicates were removed when present. Titles and abstracts were screened to identify relevant studies, followed by full-text assessment according to predefined inclusion and exclusion criteria described above.

Studies were included if they (i) investigated resveratrol or its derivatives (e.g., polydatin) and (ii) evaluated their biological effects in OSCC experimental models. Non-experimental publications (reviews, editorials, commentaries), studies not involving OSCC, and articles lacking direct evaluation of the compounds of interest were excluded. All eligible articles were analyzed in full to extract details regarding experimental models, concentrations, molecular targets, functional outcomes, and mechanistic insights relevant to OSCC.

## 3 Resveratrol

Resveratrol (3,5,4′-trihydroxystilbene) is a naturally occurring stilbenoid phytoalexin produced by several plant species as part of their defense mechanism against environmental stressors, including fungal invasion [15,16,17]. Its biosynthesis involves the enzyme resveratrol synthase, which catalyzes a condensation reaction between one molecule of p-coumaroyl-CoA and three molecules of malonyl-CoA through the shikimate-derived phenylpropanoid pathway. The activity of this enzyme is tightly regulated by various stress-related signaling compounds and elicitors that modulate plant secondary metabolism [18,19,20].

**Figure 1:**
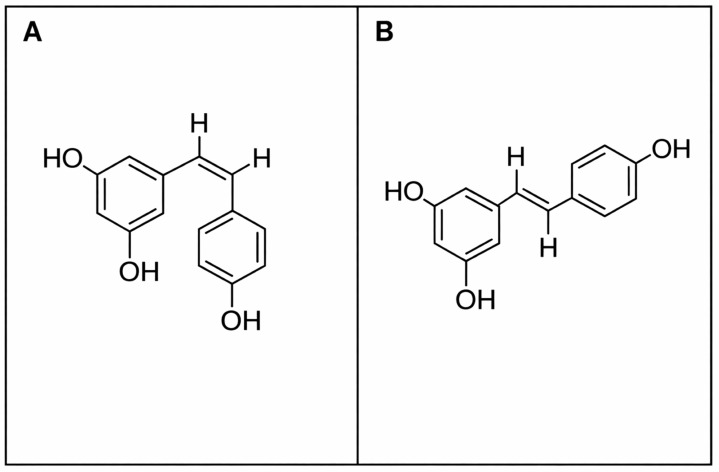
Chemical structure of cis-resveratrol (**A**) and trans-resveratrol (**B**).

Two geometric isomers of resveratrol have been identified: *trans* and *cis* (Fig. 1). The *trans* configuration exhibits greater thermodynamic stability and biological efficacy than the *cis* form, although it may undergo photoisomerization under ultraviolet light exposure. In plants, resveratrol can also exist in glycosylated derivatives, whose biological activity and absorption characteristics differ from those of the aglycone. Hydrolytic enzymes, such as glucosidases within the gastrointestinal tract, influence the metabolic conversion and bioavailability of these conjugated forms [21].

Among edible sources, skins and seeds of grapes represent the predominant dietary reservoir of resveratrol, and their content increases in response to abiotic and biotic stress conditions such as low temperature or fungal infection [22,23,24]. Red wine and grape juice generally contain higher levels of *trans*-resveratrol compared to other food products like cocoa or chocolate [25,26]. Extraction procedures employing alcoholic solvents have proven efficient in isolating resveratrol from plant matrices [27].

Beyond its physiological role in plants, resveratrol has been extensively investigated for its pharmacological potential [28,29,30]. It exhibits notable cytotoxicity toward malignant cells, primarily through mechanisms involving apoptosis induction [15,31]. Moreover, oligomeric forms of resveratrol display diverse bioactivities and are typically confined to specific plant taxa, indicating evolutionary specialization within polyphenolic secondary metabolism [32,33].

Studies in both experimental models and animals have characterized the absorption profile of resveratrol, demonstrating that the compound is generally taken up efficiently after oral administration [34]. Despite this relatively high degree of intestinal uptake, circulating levels of unchanged resveratrol are consistently reported to be very low, typically remaining within the nanomolar range [35]. This discrepancy between absorption and systemic availability is largely attributed to the rapid metabolic processing of the compound. Owing to its structural complexity and physicochemical properties, trans-resveratrol undergoes extensive first-pass metabolism in the intestinal epithelium and liver, resulting in an overall oral bioavailability estimated at roughly 10–15% [35]. From a translational perspective, these pharmacokinetic constraints argue against systemic monotherapy and instead support development strategies that maximize local oral exposure (e.g., mouthwash, gel, mucoadhesive patches, or cyclodextrin-based delivery) and/or the use of stabilized derivatives with improved metabolic resistance.

The entry of resveratrol into intestinal epithelial cells occurs via passive diffusion or specialized transport systems located in the apical membrane. Once inside the enterocyte, it is promptly subjected to conjugation reactions, mainly glucuronidation and sulfation, yielding a variety of resveratrol glucuronides and sulfates [36,37]. A major fraction of the ingested compound, however, escapes early metabolism and reaches the colon largely unmodified where the resident microbiota convert resveratrol into an array of smaller phenolic metabolites, which are subsequently absorbed through the portal circulation [38]. After entering the liver, these microbial and intestinal metabolites are further processed through additional conjugation steps, including methylation, sulfation, and glucuronidation, before being released into the bloodstream and delivered to peripheral tissues. Although these metabolites circulate widely, the biological activity of many conjugated forms is substantially reduced compared to the parent molecule. This limited activity has been suggested to account for the modest effects of orally administered resveratrol on commonly assessed biological markers, including phase I/II detoxification enzymes, total antioxidant capacity, and vitamin E homeostasis [39].

Metabolites that are not utilized by peripheral tissues may be secreted back into the intestinal lumen via bile or eliminated in urine. Notably, ATP-binding cassette (ABC) membrane transporters play a central role in returning conjugated metabolites to the gut, while the unconjugated parent compound is generally not a substrate for these efflux systems. This preferential export of metabolites contributes significantly to the low systemic exposure to free resveratrol [40].

Structural modifications, particularly acetylation of hydroxyl groups, have been explored to improve these pharmacokinetic limitations. Acetylated derivatives exhibit greater metabolic stability because they are less prone to phase II conjugation, and their increased hydrophobicity facilitates more efficient membrane penetration compared with native resveratrol. Several studies have also highlighted that ABC transporters represent a major rate-limiting step in resveratrol absorption, as they actively secrete its metabolites into the intestinal lumen, thereby influencing the distribution of these compounds across different tissues [41]. After administration, resveratrol and its derivatives have been detected in multiple organs including the kidney, liver, spleen, stomach, small intestine, lung, heart, and even the brain with the highest levels frequently observed in pulmonary and cardiac tissues [40]. These data support a rational translational perspective focused on prodrug/derivative optimization (e.g., acetylated forms) and delivery systems designed to bypass transporter-driven efflux, with oral mucosal concentration–response relationships as a key development endpoint.

## 4 Oral Squamous Cell Carcinoma

OSCC represents the predominant malignant tumor of the oral cavity and is classified within the broader category of head and neck squamous cell carcinomas (HNSCCs). Because the vast majority of oral malignancies arise from the stratified squamous epithelium, OSCC accounts for nearly all cancers originating in this anatomical region [42,43]. Globally, OSCC ranks among the most common solid tumors, with hundreds of thousands of new cases diagnosed each year, and its clinical relevance is underscored by a persistently high mortality rate [44]. The disease is marked by considerable biological aggressiveness, a strong propensity for regional or distant dissemination, and an overall 5-year survival that remains close to 50% despite therapeutic advances [45]. Tumors may originate from any mucosal site within the oral cavity including the tongue, floor of the mouth, gingiva, buccal mucosa, lip, or palate, with lesions of the tongue constituting the most frequently encountered subtype [46]. OSCC mainly affects men, and its incidence increases with age, with most patients receiving a diagnosis between the fifth and seventh decade of life [47,48]. Despite ongoing public health initiatives, a large proportion of cases continue to be detected at advanced or metastatic stages, whereas only a minority of patients present with early-stage, localized disease at the time of diagnosis [49,50,51]. Although OSCC is encountered worldwide, its incidence is particularly elevated in developing regions, where socioeconomic disparities, limited access to healthcare, and cultural practices involving tobacco and alcohol contribute substantially to cancer risk. The burden of disease is especially pronounced in several South and Southeast Asian countries and in certain Latin American and Pacific regions [52]. Geographic variation in incidence and mortality highlights the strong influence of environmental exposure, lifestyle patterns such as tobacco and alcohol consumption, and public health infrastructure on disease development and patient outcomes [53]. Chronic exposure of oral epithelial cells to mutagenic agents initiates a cascade of inflammatory and dysplastic changes, with precancerous lesions such as leukoplakia and erythroplakia representing key intermediates in the progression toward malignant transformation [54].

While tobacco use and alcohol consumption remain the major etiological factors, infection with oncogenic human papillomavirus (HPV) has emerged as an additional driver of a subset of OSCC cases. HPV-associated tumors tend to arise in younger individuals and are often characterized by a more favorable clinical trajectory, including improved survival and enhanced responsiveness to chemoradiation, compared to HPV-negative tumors [55,56].

Analogously to many solid malignancies, late diagnosis is a central challenge in OSCC management. Early detection is critical for reducing morbidity and mortality, yet diagnostic delays remain common and are frequently attributed either to misinterpretation of early lesions by clinicians or to patient-related factors such as neglect of initial symptoms [42]. Standard diagnostic evaluation typically involves clinical inspection and palpation of the oral cavity followed by tissue biopsy for histopathological confirmation [57]. Prognosis is strongly influenced by tumor stage at presentation as well as modifiable lifestyle factors and comorbidities.

Therapeutic decision-making is guided primarily by tumor extent. Individuals diagnosed with limited, early-stage disease are generally managed with surgical resection, with adjuvant radiotherapy or concurrent chemoradiotherapy administered selectively based on postoperative histopathological findings. Such patients often achieve comparatively favorable outcomes and exhibit substantially higher cure rates than those with more advanced disease [58]. In contrast, patients with locally advanced OSCC typically undergo primary surgical removal of the tumor followed by adjuvant chemotherapy and radiation, whereas neoadjuvant chemotherapy may be employed in cases deemed unresectable [59].

Resistance to chemotherapy is a major challenge for the treatment of several types of cancers, including OSCC, since it can significantly worsen the outcome of cancer patients [60,61]. Although several cytotoxic agents including platinum compounds (cisplatin, carboplatin), antimetabolites (5-Fluorouracil, methotrexate), taxanes (docetaxel), bleomycin and hydroxyurea are routinely used in the management of metastatic or recurrent disease, tumors frequently acquire resistance, leading to disease relapse and further dissemination [62,63,64]. In addition, both extensive surgical procedures and organ-preserving chemoradiation can result in long-term functional impairment and significant declines in quality of life. These limitations highlight the urgent need for innovative therapeutic strategies capable of improving clinical outcomes while reducing treatment-related morbidity [65]. These challenges have encouraged the investigation of novel antineoplastic strategies, including the use of resveratrol, a natural molecule capable of modulating tumor biology and potentiating the response to standard chemotherapy. Within current OSCC care pathways, the most plausible near-term role for resveratrol is as a treatment sensitizer or dose-sparing adjunct to standard modalities, potentially improving local control while limiting cumulative toxicity in multimodal regimens.

## 5 Resveratrol in OSCC

### 5.1 Antiproliferative Effects, Cell-Cycle Regulation and Apoptosis

#### 5.1.1 Effects on Cell Proliferation and Cell-Cycle Arrest

Berta et al. were the first group that investigated the effect of resveratrol on OSCC. In this study, the authors investigated the anticancer potential of resveratrol against oral carcinogenesis using both *in vitro* and *in vivo* models, and assessed whether complexing the molecule with 2-hydroxypropyl-β-cyclodextrin (HPβCD) could enhance its biological activity. *In vitro*, they employed the DMBA-induced hamster OSCC cell line HCPC I and demonstrated that resveratrol exerted a clear dose- and time-dependent antiproliferative effect, which became significantly more potent when the compound was delivered in HPβCD-based cream or mouthwash formulations. *In vivo*, the authors used the classic Syrian hamster cheek pouch model of DMBA-induced oral carcinogenesis and showed that topical application of resveratrol markedly reduced the incidence, multiplicity, and size of oral preneoplastic lesions and exophytic tumors. These effects were most pronounced when resveratrol was administered in the HPβCD mouthwash, which consistently outperformed both the HPβCD cream and resveratrol in ethanol. Histological analyses confirmed a decreased frequency and severity of malignant lesions in treated animals, and HPLC measurements revealed substantially higher mucosal concentrations of resveratrol following HPβCD-based delivery. Overall, the study demonstrated that resveratrol can effectively suppress the progression of DMBA-induced oral lesions and that its efficacy is significantly enhanced by complexation with HPβCD, which improves solubility, mucosal uptake, and local bioavailability [66]. This delivery approach is highly relevant translationally, as topical oral formulations could be evaluated in patients with potentially malignant oral disorders or high-risk surgical margins, with mucosal drug levels and modulation of EMT/apoptosis markers serving as early pharmacodynamic readouts.

**Figure 2:**
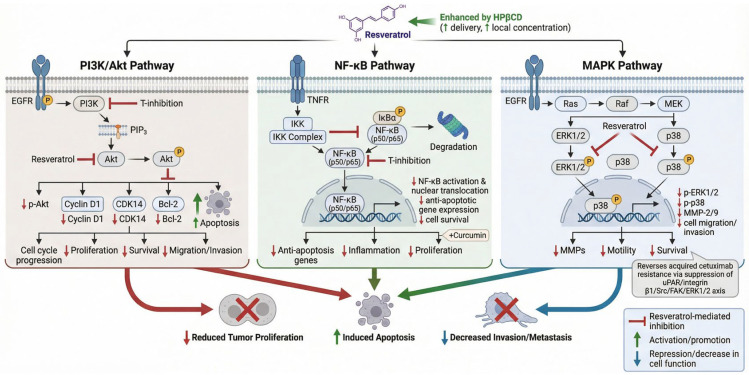
Resveratrol-mediated modulation of oncogenic signaling pathways. In the PI3K/Akt pathway, resveratrol inhibits Akt phosphorylation, leading to reduced expression of Cyclin D1, CDK14, and Bcl-2, thereby suppressing cell cycle progression, proliferation, survival, and migration/invasion while promoting apoptosis. In the NF-κB pathway, resveratrol blocks IKK complex activity and NF-κB (p50/p65) nuclear translocation, resulting in decreased anti-apoptotic gene expression, inflammation, and proliferation. In the MAPK pathway, resveratrol inhibits ERK1/2 and p38 phosphorylation downstream of EGFR/Ras/Raf/MEK signaling, reducing MMP-2/9 expression, cell motility, and survival, and contributing to decreased invasion and metastasis. Additionally, resveratrol may reverse acquired cetuximab resistance via suppression of the uPAR/integrin β1/Src/FAK/ERK1/2 axis.

Yu et al. evaluated the anticancer effects of resveratrol in three OSCC cell lines (SCC-VII, SCC-25, and YD-38) and demonstrated that the compound exerts strong antiproliferative and pro-apoptotic activity (Fig. 2). Resveratrol significantly reduced cell viability in a concentration- and time-dependent manner, with IC_50_ values ranging from 0.5 to 1.0 μg/mL after 48 h of exposure. Analysis of cell-cycle distribution showed that resveratrol induced a pronounced G2/M arrest, accompanied by a marked reduction in the proportion of cells in the G1 phase. At the molecular level, resveratrol increased the expression of key regulators associated with G2/M transition, including phosphorylated cdc2 (Tyr15), cyclin A2, cyclin B1, and the inhibitory kinase Myt1, while also enhancing γ-H2AX, indicating activation of DNA damage signaling pathways. Furthermore, Annexin-V–based assays confirmed that resveratrol strongly promotes apoptosis in all tested OSCC cell lines, with apoptotic populations increasing substantially with longer treatment. Collectively, these findings highlighted the robust antitumor role of resveratrol, demonstrating its ability to inhibit OSCC cell proliferation by triggering G2/M arrest and apoptotic cell death, supporting its potential as a therapeutic candidate in oral cancer management [67].

Hayashi et al. identified tripartite motif family-like 2 (TRIML2) as a previously uncharacterized regulator of OSCC growth and demonstrated that resveratrol functions as a pharmacological inhibitor of this oncogenic protein. TRIML2 was found to be markedly overexpressed in OSCC cell lines and primary tumor tissues, and its knockdown suppressed proliferation by inducing G1-phase arrest associated with reduced CDK2, CDK4, and cyclin D1 levels together with increased p21^Cip1^ and p27^Kip1^ expression. Importantly, resveratrol reproduced all key molecular and phenotypic effects of TRIML2 silencing: treatment with resveratrol significantly downregulated TRIML2 mRNA and protein expression, impaired OSCC cell growth, and triggered a robust G1 arrest accompanied by the same pattern of cyclin/CDK downregulation and CDK-inhibitor upregulation observed in TRIML2-deficient cells. These findings indicate that resveratrol exerts its antitumor activity in OSCC at least in part by targeting TRIML2, thereby restoring p21-mediated checkpoint control and halting tumor cell proliferation. Overall, the study revealed resveratrol as a promising therapeutic candidate capable of interfering with a newly identified pathway essential for OSCC progression [68].

#### 5.1.2 Induction of Apoptosis and Ferroptosis

Mao et al. reported that resveratrol powerfully suppresses OSCC progression by inducing ferroptosis through the p53/SLC7A11 regulatory axis. Treatment of CAL-27 and SCC-9 cells with resveratrol significantly reduced viability, colony formation, and migratory and invasive capacity, and it produced a clear G1-phase arrest. Mechanistically, resveratrol promoted the nuclear accumulation of p53, which in turn repressed the transcription of SLC7A11, a transporter essential for cystine uptake and glutathione synthesis. This shift impaired the antioxidant defenses of OSCC cells, leading to increased intracellular iron, enhanced reactive oxygen species, reduced glutathione levels, and decreased GPX4 expression, collectively defining a ferroptotic response. Inhibition or knockdown of p53 weakened the ability of resveratrol to trigger ferroptosis and to restrict malignant behaviors, while silencing SLC7A11 restored these effects even when p53 activity was suppressed, confirming SLC7A11 as a critical downstream mediator. These findings showed that resveratrol acts as a potent inducer of ferroptosis in OSCC by activating p53 and suppressing SLC7A11, revealing a therapeutic mechanism that simultaneously impairs redox homeostasis and tumor aggressiveness [69]. Kim et al. examined the anticancer activity of resveratrol in three human OSCC cell lines (CAL27, SCC15, and SCC25) with the aim of elucidating its mechanistic effects on apoptosis, migration, invasion, and epithelial–mesenchymal transition (EMT). Resveratrol significantly decreased cell viability in a clear time- and dose-dependent manner, with CAL27 cells showing the greatest sensitivity. The compound robustly induced apoptosis, as demonstrated by nuclear condensation, increased Annexin V–positive populations, loss of mitochondrial membrane potential, and activation of mitochondrial apoptotic signaling. Mechanistically, resveratrol triggered the intrinsic apoptotic pathway by upregulating the pro-apoptotic proteins Bax and Bak, downregulating the anti-apoptotic proteins Bcl-2 and Bcl-XL, promoting cytochrome c release, and activating downstream caspase-9, caspase-3, ICAD, and PARP cleavage. In addition to its pro-apoptotic effects, resveratrol markedly inhibited OSCC cell migration and invasion, as shown by wound-healing and transwell assays. These anti-metastatic effects were accompanied by a strong suppression of EMT, reflected by increased E-cadherin expression and reduced levels of N-cadherin, Snail, Slug, and Smad2/3. Taken together, this work demonstrated that resveratrol exerts potent antitumor effects in OSCC by simultaneously activating mitochondrial apoptosis and suppressing EMT-associated motility, supporting its potential as an effective therapeutic agent in oral cancer [70].

#### 5.1.3 Regulation of Oncogenic Axes Controlling Proliferation

Zhang et al. showed that resveratrol significantly restrains the progression of OSCC by disrupting the NORAD/IGF2BP2/PDK1 signaling cascade, a pathway closely linked to uncontrolled proliferation and enhanced motility in malignant cells. Resveratrol reduced the viability of CAL-27, KB, and SCC-25 cells in a concentration-dependent manner, with CAL-27 cells displaying the highest sensitivity. In CAL-27 cells, resveratrol triggered cell-cycle arrest at the G2/M phase and, at higher concentrations, at both the S and G2/M phases, indicating interference with key checkpoints that regulate DNA synthesis and mitotic entry. At the molecular level, resveratrol lowered the expression of the long noncoding RNA NORAD, the RNA-binding protein IGF2BP2, and the kinase PDK1, while also suppressing several associated downstream regulators of proliferation and survival. Functional experiments further demonstrated that silencing NORAD or IGF2BP2 reduced OSCC cell proliferation, migration, and invasion, and manipulating PDK1 expression confirmed that this kinase contributes to malignant behavior. Altogether, the findings indicated that resveratrol acts through coordinated inhibition of NORAD, IGF2BP2, and PDK1, leading to impaired cell growth and reduced invasive potential, and support the involvement of this regulatory axis in mediating resveratrol’s anticancer activity in OSCC [71].

### 5.2 Inhibition of Migration, Invasion and Epithelial–Mesenchymal Transition

Zhou et al. examined the anti-metastatic properties of resveratrol in the OSCC cell line KB, focusing specifically on its effects on cell adhesion, migration, and invasion. Using *in vitro* assays, they exposed KB cells to increasing concentrations of resveratrol and assessed adhesion through an MTT-based matrigel assay, while migration and invasion were evaluated using transwell chambers, the latter incorporating a matrigel coating to simulate extracellular matrix barriers. The results demonstrated that resveratrol significantly impaired the metastatic phenotype of KB cells: at 100 μM, the compound reduced cell adhesion by approximately 50–58% within 1–2 h, suppressed migratory activity by nearly 44–48%, and inhibited invasive capacity by about 38% compared to untreated controls. These findings indicated that resveratrol can effectively disrupt key steps of the metastatic cascade in OSCC cells, suggesting a potential role as anticancer and anti-metastatic agent (Fig. 2) [72].

Kim et al. demonstrated that resveratrol exerts a strong inhibitory effect on the invasive and metastatic behavior of OSCC cells by disrupting the Rab-coupling protein (RCP) signaling axis, a pathway identified as a major driver of OSCC progression. RCP enhances OSCC invasion through the induction of EMT, mediated by increased expression of Zeb1 and the protease MT1-MMP, and through the activation of β1-integrin, EGFR, and β-catenin signaling. Importantly, resveratrol was shown to block this entire pro-invasive program, suppressing RCP-induced β1-integrin recycling, inhibiting EGFR activation, preventing β-catenin nuclear signaling, and strongly reducing the expression of Zeb1 and MT1-MMP. Functionally, these molecular effects resulted in a marked reduction in OSCC cell motility, migration, and invasiveness across multiple experimental models. Overall, the findings highlighted that resveratrol acts as a potent antagonist of RCP-dependent EMT and invasion, reinforcing its potential therapeutic value as a natural compound capable of targeting metastatic mechanisms in OSCC [73].

Chang et al. demonstrated that resveratrol effectively suppresses the metastatic behavior of cisplatin-resistant OSCC cells, highlighting its potential as an adjuvant therapy in drug-resistant disease. Using the cisplatin-resistant CAR cell line, they showed that resveratrol, at non-cytotoxic concentrations, significantly inhibited cell migration and invasion in a dose-dependent manner. Importantly, the compound exerted its anti-metastatic effects by downregulating the phosphorylated forms of ERK and p38, two MAPK signaling components commonly associated with enhanced motility and metastatic capacity in resistant cancer cells. Resveratrol also suppressed the expression of matrix metalloproteinase (MMP)-2 and MMP-9, key MMPs required for extracellular matrix degradation and cancer cell invasion. Notably, these effects occurred rapidly, within 24 h, and at doses that did not impair CAR cell viability, indicating a specific action on metastatic signaling rather than general toxicity. Overall, the study demonstrated that resveratrol can directly counteract the metastatic phenotype of cisplatin-resistant OSCC cells by targeting MAPK phosphorylation and MMP expression, suggesting that resveratrol could be explored as an anti-metastatic adjunct in platinum-resistant OSCC, potentially targeting dissemination-related pathways even at non-cytotoxic concentrations, which may be achievable locally in the oral cavity [74].

Min et al. investigated the capacity of resveratrol to counteract EMT in OSCC cells exposed to *Fusobacterium nucleatum*, a bacterium increasingly recognized as a promoter of EMT and metastatic behavior in OSCC. Using HSC-3 OSCC cells co-cultured with *F. nucleatum*, the authors confirmed that bacterial exposure enhances cell motility and induces EMT-associated molecular alterations, including upregulation of the transcription factor SNAI1 and loss of the epithelial marker E-cadherin. Resveratrol demonstrated a significant inhibitory effect on this process: at a non-bactericidal concentration of 10 μM, it suppressed *F. nucleatum*–induced cell migration and effectively reversed EMT-related gene and protein changes. In particular, resveratrol restored E-cadherin expression and reduced SNAI1 levels, indicating a direct impact on key regulators of EMT. These findings highlighted resveratrol as a potent modulator of microbially induced EMT in OSCC, suggesting that it may help mitigate metastasis-associated phenotypes driven by pathogenic components of the tumor microenvironment [75].

### 5.3 Modulation of Oncogenic Signaling, Tumor Microenvironment, and Therapeutic Response

#### 5.3.1 Reversal of Drug Resistance and Therapeutic Sensitization

Uzawa et al. investigated the molecular basis of acquired cetuximab resistance in OSCC and demonstrated that resveratrol can effectively reverse this resistance by targeting a uPAR-dependent signaling axis. Gene-expression profiling of cetuximab-resistant OSCC cell lines (SAS-R, Sa3-R, HSC-3-R) revealed marked upregulation of urokinase-type plasminogen activator receptor (uPAR), which activated an integrin β1/Src/FAK/ERK1/2 circuit independent of EGFR mutations. Functional experiments confirmed that resistant cells displayed enhanced ERK1/2 phosphorylation and increased proliferation despite cetuximab exposure. Importantly, resveratrol potently downregulated uPAR, integrin β1, and phosphorylated ERK1/2, mirroring the effects of shRNA-mediated uPAR silencing and significantly reducing viability of cetuximab-resistant cells *in vitro*. In xenograft models, resveratrol treatment markedly suppressed tumor growth and, when combined with cetuximab, produced an almost complete inhibition of tumor progression without detectable toxicity. Immunohistochemistry further showed that resveratrol restored cetuximab sensitivity by reducing uPAR/integrin β1 signaling and lowering ERK1/2 activation in resistant tumors. This study demonstrated that resveratrol acts as a functional inhibitor of the uPAR-driven bypass pathway that mediates cetuximab resistance, thereby re-sensitizing OSCC cells to anti-EGFR therapy and highlighting its potential as a valuable adjunct in targeted treatment strategies [76]. This mechanism highlights a potential translational niche in biomarker-guided salvage therapy: patients with uPAR/integrin-driven bypass activation may represent a subgroup in whom resveratrol-based adjunct strategies could restore sensitivity to EGFR blockade.

Atienzar et al. evaluated the *in vitro* antitumor effects of resveratrol alone and in combination with ionizing radiation in the human OSCC cell line PE/CA-PJ15. Resveratrol was administered at concentrations ranging from 5 to 100 μM, and cells were irradiated with single doses of 1, 2.5, or 5 Gy to assess potential radiosensitizing effects. Across multiple time points (24, 48, and 72 h), resveratrol exerted a clear dose-dependent cytotoxic action, with the highest concentration (100 μM) producing the most pronounced reduction in cell viability and the strongest induction of apoptosis at all radiation doses. Cell cycle analysis demonstrated that 50 and 100 μM resveratrol significantly altered phase distribution by increasing S-phase arrest at the expense of the G0/G1 and G2/M populations, a shift that can enhance cellular sensitivity to radiation. Moreover, migration assays revealed that resveratrol markedly impaired the motility of PE/CA-PJ15 cells, with substantial inhibition observed particularly at 25 and 100 μM when combined with irradiation. Overall, the study showed that resveratrol not only reduces OSCC cell viability, promotes apoptosis, and disrupts cell cycle progression but also acts synergistically with radiation to further suppress migration and enhance therapeutic cytotoxicity, suggesting its potential utility as a radiosensitizing adjunct in OSCC management [77]. If confirmed *in vivo*, the radiosensitizing profile of resveratrol may have practical implications for organ-preservation strategies and for intensification of local control in radioresistant disease subsets, provided that clinically achievable mucosal concentrations can be attained through topical or loco-regional delivery.

Tomikoshi et al. evaluated whether benzoxazinotropone (BOT), a heterocyclic compound with known tumor-selective and anti-inflammatory activity, could potentiate the cytotoxic effects of doxorubicin, curcumin, and resveratrol against human OSCC cells while sparing normal oral cells. Using a panel of OSCC lines (Ca9-22, HSC-2, HSC-3, HSC-4) and multiple normal oral cell types, they first confirmed that BOT triggers apoptosis in HSC-2 cells, as shown by dose-dependent activation of caspase-3 and cleavage of PARP (page 3). BOT was then combined with each agent and cytotoxicity quantified via MTT assays and synergy assessed using CompuSyn. Importantly, resveratrol displayed intrinsic tumor-selective cytotoxicity, with a tumor-selectivity index comparable to curcumin, and its combination with BOT yielded both additive and synergistic effects depending on the concentration. Synergy was most evident at lower resveratrol doses (3.1–6.3 μM), significantly enhancing growth inhibition of HSC-2 cells beyond either compound alone. At higher concentrations (12.5–200 μM), BOT and resveratrol acted additively. Notably, BOT did not increase the cytotoxicity of resveratrol toward normal oral epithelial cells, suggesting a preserved therapeutic window. Overall, the findings underscore that resveratrol not only exerts direct anticancer activity against OSCC cells but also serves as a synergistic partner to BOT, enhancing apoptotic responses selectively in tumor cells and supporting its potential role in combination therapies [78].

#### 5.3.2 Modulation of the Tumor Microenvironment

Li et al. reported that resveratrol markedly reduced the invasive and migratory behavior of OSCC cells by acting on tumor-associated macrophages (TAMs) rather than directly on the carcinoma cells themselves. Using a macrophage model conditioned by OSCC-derived medium, the study demonstrated that resveratrol is able to reprogram TAMs from an M2, tumor-promoting phenotype into an M1, tumor-restraining state. This shift was supported by increased expression of M1-associated cytokines and reduced production of M2-related markers. Resveratrol applied either after TAM induction or concurrently with OSCC-conditioned medium consistently generated a macrophage population that impaired OSCC cell migration and invasion. Mechanistically, resveratrol inhibited activation of the Syk signaling pathway in TAMs, and pharmacological suppression of Syk produced similar alterations in macrophage phenotype and comparable reductions in OSCC cell aggressiveness. These findings showed that resveratrol indirectly restrains OSCC progression by remodeling the immune microenvironment, specifically by steering macrophages toward an antitumor phenotype through inhibition of Syk-dependent polarization [79]. The ability to reprogram TAM polarization may suggest translational compatibility with immunotherapy-oriented strategies, raising the hypothesis that resveratrol may support immune-permissive remodeling of the OSCC microenvironment and could be evaluated alongside checkpoint blockade in preclinical combination models.

Pilankar et al. investigated the effects of an orally administered resveratrol–copper (R–Cu) formulation on the tumor microenvironment of patients with advanced OSCC. The therapeutic concept was based on the ability of resveratrol, when combined with trace amounts of copper, to generate oxygen radicals capable of degrading cell-free chromatin particles (cfChPs), which act as potent inducers of multiple cancer hallmarks. Two weeks of R–Cu administration resulted in a substantial reduction in cfChPs within the tumor microenvironment, accompanied by a broad suppression of biomarkers associated with key malignant traits, including uncontrolled proliferation, inflammation, angiogenesis, invasion, resistance to cell death, and immune evasion. The treatment also led to the downregulation of several major immune checkpoint proteins present in tumor-infiltrating lymphocytes. Notably, these effects were most pronounced at the lowest R–Cu doses, suggesting that minimal quantities of resveratrol combined with copper are sufficient to elicit significant biological responses. Overall, the findings provided first-in-human evidence that resveratrol, through its pro-oxidant activity in the R–Cu complex, can neutralize cfChPs and suppress a wide array of oncogenic and immunosuppressive pathways in OSCC, highlighting a potentially novel and indirect therapeutic mechanism centered on modifying the tumor microenvironment rather than directly targeting cancer cells [80].

#### 5.3.3 Optimization Strategies: Structural Derivatives and Analogues

Uesawa et al. synthesized sixteen 3-styryl-2H-chromene derivatives—structurally related to resveratrol—and systematically evaluated their cytotoxicity and tumor selectivity against four human OSCC cell lines (Ca9-22, HSC-2, HSC-3, HSC-4) and three normal oral cell types, complemented by additional assays using human oral keratinocytes and gingival epithelial cells. Using MTT-based viability testing, the researchers determined 50% cytotoxic concentration (CC_50_) values and calculated tumor-selectivity indices (TS) and potency-selectivity expression (PSE) scores to compare therapeutic potential. Several chromenes exhibited markedly higher tumor selectivity than resveratrol itself, with compound [12]—bearing a methoxy group at the 7-position of the chromene ring and a chlorine at the 4′-position of the styryl phenyl ring—demonstrating the strongest anticancer profile, achieving CC_50_ values around 4–5 μM in OSCC cells and exceptionally high TS values exceeding those of doxorubicin and 5-fluorouracil. Conversely, all derivatives lacked anti-HIV activity, as indicated by selectivity indices below 1. Quantitative structure–activity relationship (QSAR) analyses further identified molecular descriptors related to conformation, hydrophobicity, surface properties, and flexibility as determinants of cytotoxicity and tumor specificity. Overall, the study demonstrates that specific chromene derivatives can surpass resveratrol in selective toxicity toward OSCC cells, highlighting structural features that may guide the development of more potent and tumor-targeted analogues [81].

#### 5.3.4 Translational and Epidemiological Evidence

In a population-based case–control study, Lee et al. investigated whether a dietary pattern rich in the phytochemicals lignans, quercetin and resveratrol was associated with a reduced risk of oesophageal cancer. Using data from 181 oesophageal adenocarcinoma (OAC) cases, 158 oesophageal squamous cell carcinoma (ESCC) cases, 255 gastro-oesophageal junction adenocarcinoma (JAC) cases, and 806 matched controls, dietary exposures were assessed through validated food-frequency questionnaires reflecting intake 20 years before diagnosis. Reduced-rank regression was applied to derive a dietary pattern that captured variation in the intake of the three phytochemicals, revealing that higher pattern scores were mainly driven by consumption of tea, wine, lettuce, mixed vegetables, tomatoes, and whole-grain bread, whereas milk intake contributed negatively. Logistic regression analyses demonstrated a strong, dose-dependent inverse association between adherence to this phytochemical-rich dietary pattern and the risk of all oesophageal cancer subtypes. Individuals in the highest dietary pattern quintile had substantially lower adjusted odds ratios compared with those in the lowest quintile—including a 76% reduction for OAC, 69% for ESCC, and 51% for JAC—after adjustment for established risk factors such as smoking, alcohol consumption, BMI, gastro-oesophageal reflux, and Helicobacter pylori infection. These findings indicate that a diet naturally enriched in lignans, quercetin and resveratrol may exert a protective effect against the development of multiple forms of oesophageal cancer. Although this study was focused on ESCC, OSCC and ESCC share several biological and clinical characteristics because they arise from similar squamous epithelial cells and are influenced by overlapping risk factors [82]. Both cancers are strongly associated with tobacco and alcohol use, exhibit early genetic alterations such as TP53 mutations, and often develop from precancerous dysplastic lesions [53,83,84].

### 5.4 Resveratrol Derivatives and Related Stilbenes

Martano et al. investigated the clinical relevance of aryl hydrocarbon receptor (AHR) signaling in OSCC and assessed the biological effects of polydatin, a naturally occurring stilbenoid and direct precursor of resveratrol, on AHR-related pathways. Immunohistochemical analysis of 25 human OSCC specimens revealed that AHR, its downstream target CYP1A1, and the chaperone protein HSP-90 were markedly upregulated in tumor tissues, with expression levels increasing progressively from low- to high-grade carcinomas. To explore whether polydatin could modulate this pathway, the authors treated CAL27 OSCC cells with increasing concentrations of the compound and observed a clear, dose-dependent reduction in AHR, CYP1A1, and HSP-90 protein expression after 24 h. Polydatin also inhibited CAL27 cell proliferation with an IC_50_ of approximately 20 μM, demonstrating intrinsic antitumor activity linked to suppression of AHR signaling. Molecular docking simulations further supported a direct interaction between polydatin and the AHR ligand-binding domain, suggesting that the glycosylated structure of polydatin does not hinder—and may even stabilize—binding relative to its resveratrol aglycone. Overall, the study indicated that polydatin, as a resveratrol precursor with improved bioavailability, can effectively downregulate AHR signaling and reduce OSCC cell growth, supporting its potential use in oral cancer prevention or as an adjunct to antineoplastic therapy [85].

Bang et al. also examined the anticancer properties of polydatin in OSCC cell lines and demonstrated that it exerts potent pro-apoptotic and anti-metastatic effects. Polydatin significantly reduced cell viability and proliferation in Ca9-22 and CAL27 cells, showing preferential cytotoxicity toward OSCC compared with non-malignant keratinocytes. Mechanistically, polydatin activated the intrinsic mitochondrial apoptotic pathway, promoting cytochrome c release, downregulation of the anti-apoptotic protein Bcl-2, upregulation of Bax, and subsequent cleavage of caspase-3 and PARP. In addition to apoptosis, polydatin induced autophagic cell death, increasing ATG5 and LC3 expression. The compound also markedly impaired OSCC cell motility and invasiveness, suppressing epithelial–mesenchymal transition by upregulating E-cadherin and downregulating N-cadherin and the transcriptional regulators Snail and Slug. Collectively, these findings showed that polydatin, reflecting the known anticancer activity of its aglycone resveratrol, can concurrently trigger mitochondrial apoptosis, stimulate autophagy, and inhibit EMT-mediated metastatic behavior, supporting its potential as a resveratrol-derived therapeutic candidate for OSCC [86].

Hsieh et al. investigated the antimetastatic properties of pinostilbene hydrate (PSH), a naturally occurring methylated derivative of resveratrol, in three human OSCC cell lines (SCC-9, SAS, and HSC-3). Methylated resveratrol analogues such as PSH are known to exhibit improved stability and bioactivity compared with resveratrol itself, and this work specifically explored whether PSH could inhibit OSCC cell migration and invasion. The compound exhibited no significant cytotoxic effects at concentrations up to 80 μM, allowing the assessment of metastasis-related behaviors independently of cell death. Using wound-healing and transwell assays, the authors demonstrated that PSH markedly reduced cell motility, migration, and invasion in all three OSCC models in a dose-dependent manner. Mechanistically, PSH significantly suppressed both enzymatic activity and protein expression of MMP-2, a key mediator of extracellular matrix degradation and metastatic progression. Further analysis revealed that PSH inhibited phosphorylation of ERK1/2 and p38 MAPK in SCC-9 and SAS cells, indicating that its antimetastatic effects are mediated through downregulation of MAPK signaling. The inhibitory effects on migration and MMP-2 activity were even stronger when PSH was combined with ERK or p38 inhibitors, confirming pathway involvement. Taken together, this work showed that the resveratrol-derived molecule PSH functions as an effective suppressor of OSCC metastatic behavior by targeting MMP-2 and MAPK-associated signaling, supporting its potential as a therapeutic or preventive agent against oral cancer metastasis [87]. From a translational perspective, resveratrol derivatives such as polydatin and methylated analogues may offer certain pharmacokinetic advantages compared with native resveratrol, including improved stability, enhanced bioavailability, and greater metabolic resistance. However, resveratrol itself remains the most extensively studied compound, with a broader body of mechanistic and experimental evidence currently available in OSCC. Therefore, while derivatives may represent promising optimization strategies, the translational potential of these compounds should be considered complementary rather than replacement approaches, pending further comparative investigation. The above-reported studies investigating the anticancer effects of resveratrol in OSCC are comprehensively summarized in Table 1.

**Table 1:** Summary of the anticancer and antimetastatic activities of resveratrol in OSCC models, detailing drug concentrations, model used, molecular markers assessed, and major findings.

Concentrations	Model Type	Type of Cells	Assays	Markers/Pathways	Results	Ref.
5–100 μM, up to 72 h	Cell lines and xenograft	HCPC I	MTT/Immunohistochemistry	Proliferation	Inhibited proliferation; enhanced by HPβCD formulation; reduced tumor burden *in vivo*	[66]
Up to 100 μM	Cell lines	KB	MTT/Transwell migration	Migration, invasion	Reduced adhesion, migration, and invasion	[72]
5–100 μM for 24–72 h	Cell lines	PE/CA-PJ15	MTT/Annexin V/Transwell migration	Cell cycle, apoptosis	S-phase arrest; enhanced radiosensitivity; reduced migration	[77]
CC_50_ evaluation	Cell lines	Ca9-22, HSC-2, HSC-3, HSC-4	MTT	Cytotoxicity, QSAR	Chromenes showed higher tumor selectivity than resveratrol	[81]
3.1–200 μM	Cell lines	Ca9-22, HSC-2, HSC-3, HSC-4	MTT/Western Blot	Caspases, PARP	Synergistic apoptosis with benzoxazinotropone	[78]
Up to 1 μg/mL (4.38 μM)	Cell lines	SCC-VII, SCC-25, YD-38	MTT/Flow cytometry/Western Blot	Cyclins, Myt1, γ-H2AX	G2/M arrest; DNA damage; apoptosis	[67]
10–500 μM	Cell lines	CAL-27, SCC-15, SCC-25	MTT/Fluorescence Microscopy/Annexin V/Western Blot	Bax, Bak, EMT markers	Apoptosis induction; EMT suppression; reduced invasion	[70]
50 μM	Cell lines	CAL-27	Real-time PCR/Western Blot/Immunohistochemistry	TRIML2 pathway	TRIML2 suppression; G1 arrest; reduced proliferation	[68]
20 μM; 100 mg/kg intraperitoneally daily	Cell lines	Cetuximab-resistant SAS-R, Sa3-R, HSC-3-R	Microarray/Real-time PCR/Western Blot/MTT/Immunohistochemistry	uPAR-integrin pathway	Reversed cetuximab resistance; inhibited ERK; restored drug sensitivity	[76]
Up to 25 μM	Cell lines	YD-9, YD-38 and YD-10B	Real-time PCR/Western Blot/Immunofluorescence/Wound healing/MTT/Transwell invasion	RCP, β1-integrin, EGFR	Blocked EMT and invasion; inhibited integrin recycling and signaling	[73]
0–75 μM	Cell lines	Cisplatin-resistant CAL-27	MTT/Transwell migration/Wound healing/Western Blot	ERK, p38, MMPs	Reduced migration; downregulated MMP-2/9; MAPK inhibition	[74]
Clinical R–Cu formulation (from 5.6 mg of R and 560 ng of Cu mg to 500 mg of R and 5 mg of Cu)	*In vivo* human subjects	OSCC patients	Immunofluorescence/Immunohistochemistry	cfChPs, immune markers	Reduced cfChPs; decreased oncogenic and immune checkpoint markers	[80]
10 μM	Cell lines	HSC-3	MTT/Wound healing/Real-time PCR/Western Blot/Immunofluorescence	EMT markers	Reversed *F. nucleatum*-induced EMT; reduced migration	[75]
10–100 μM	Cell lines	CAL-27, SCC-9	MTT/CCK-8/Transwell migration/Flow cytometry	p53/SLC7A11	Induced ferroptosis; impaired migration	[69]
50–125 μg/mL (219–548 μM)	Cell lines	CAL-27, KB, SCC-25	MTT/Flow cytometry/Western Blot/Transwell migration	NORAD/IGF2BP2/PDK1	Suppressed proliferation and invasion	[71]
0–40 μM	Cell lines	CAL27 and RAW264.7	Real-time PCR/ELISA/Western Blot/Immunofluorescence/Transwell migration/Wound healing	Syk pathway	Reprogrammed TAMs; reduced OSCC migration/invasion	[79]
0–150 μM	Cell lines	CAL-27	Immunohistochemistry	AHR, CYP1A1, HSP-90	Inhibited proliferation; downregulated AHR pathway; antitumor effects	[85]
0–0.5 mM	Cell lines	CAL27 and Ca9-22	MTT/Immunofluorescence/Wound healing/Western Blot/Real-time PCR	Bcl-2, Bax, caspase-3 and PARP, ATG5 and LC3 expression; E-cadherin, N-cadherin and transcriptional regulators Snail and Slug.	Triggered mitochondrial apoptosis, stimulated autophagy, and inhibited EMT-mediated metastatic behavior	[86]
0–80 μM	Cell lines	SCC-9, SAS, HSC-3	MTT/Wound healing/Western Blot	MMP-2, MAPKs	Inhibited migration and invasion; suppressed MMP-2 and MAPK phosphorylation	[87]

## 6 Conclusions

The body of evidence reviewed in this manuscript indicates that resveratrol exerts important anticancer effects in OSCC through a combination of antiproliferative, pro-apoptotic, anti-migratory, and microenvironment-modulating mechanisms. Across multiple *in vitro* models, resveratrol reduces cell viability, interferes with cell-cycle progression, and activates apoptosis through both mitochondrial and caspase-dependent pathways. Several studies also demonstrate that resveratrol attenuates OSCC cell migration and invasion by limiting EMT, reducing matrix-degrading enzyme activity, and downregulating signaling mediators associated with cytoskeletal remodeling and cell motility. In addition to its direct cellular effects, resveratrol influences non-malignant components of the tumor milieu, including the polarization state of TAMs and other pathways that contribute to an immunosuppressive environment. Importantly, resveratrol enhances the efficacy of established anticancer modalities such as chemotherapy, targeted therapy, and radiotherapy, and it shows the capacity to counteract resistance mechanisms involving pathways such as MAPK, uPAR-integrin signaling, and other compensatory oncogenic circuits. The identification of specific molecular targets, including TRIML2, MAGEA12, NORAD, IGF2BP2, PDK1, and components of the p53/SLC7A11 axis, further supports the notion that resveratrol affects defined regulatory networks beyond general cytotoxicity. Studies on related stilbenoids, including polydatin and methylated derivatives, suggest that structural modifications may improve biological activity or stability, although these observations remain preliminary.

## 7 Limitations

Despite these encouraging findings, several limitations must be acknowledged. The majority of the available evidence derives from *in vitro* studies. Only a limited number of investigations have evaluated resveratrol in *in vivo* OSCC models, and clinical data remain scarce. Moreover, many experimental studies employ concentrations of resveratrol that may not be readily attainable in clinical settings due to rapid metabolism, low bioavailability, and extensive conjugation. As a consequence, while *in vitro* findings provide important mechanistic insights and support biological plausibility, additional *in vivo* validation is required to determine whether these effects can be reproduced under more physiologic conditions, where factors such as tissue distribution, tumor architecture, stromal interactions, and immune components may substantially influence therapeutic response. This limitation reflects the current state of the literature and does not diminish the relevance of the mechanistic evidence summarized here, but it does define a clear priority for future investigation.

Moreover, experimental heterogeneity is substantial, with differences in resveratrol formulations, exposure times, dosing protocols, and assay endpoints, complicating direct comparison across studies. Many mechanistic insights are derived from pathway-focused analyses, leaving uncertainty regarding the interplay of multiple signaling cascades in complex *in vivo* settings. The therapeutic relevance of resveratrol metabolites also remains unclear, as some may have distinct or diminished biological activity compared with the parent compound. Finally, the potential for resveratrol to interact with standard treatments in complex ways—including synergy as well as possible context-dependent effects—requires rigorous evaluation in controlled preclinical models.

## 8 Future Perspectives

Future research should therefore include systematic pharmacokinetic and pharmacodynamic studies to determine achievable concentrations of resveratrol and its metabolites in oral tissues, with particular attention to topical or locally delivered formulations that may circumvent first-pass metabolism.

From a translational perspective, the pharmacokinetic profile of resveratrol represent a significant challenge including rapid metabolism, low systemic bioavailability, and extensive conjugation. However, OSCC offers unique opportunities for local or topical delivery strategies, including mouthwashes, gels, mucoadhesive formulations, or nanoparticle-based systems, which may allow the achievement of therapeutically relevant concentrations directly at the tumor site while minimizing systemic exposure. Such approaches could partially overcome the discrepancy between effective *in vitro* concentrations and achievable systemic levels, supporting further investigation of resveratrol as a locally delivered adjunct in OSCC management.

Importantly, well-designed *in vivo* studies are needed to establish the extent to which the molecular and functional effects observed *in vitro* translate into meaningful antitumor activity in OSCC models, including in combination with radiotherapy, chemotherapy, or targeted agents. Given the growing evidence that resveratrol modulates immune and metabolic processes relevant to OSCC progression, future studies should integrate analyses of the tumor microenvironment and explore whether resveratrol can be incorporated into combination strategies aimed at targeting both cancer cells and their supportive niches. Rational development of resveratrol derivatives or delivery systems with improved stability, tissue penetration, and target selectivity may also enhance its translational potential. Overall, the current literature supports resveratrol as a promising biologically active compound in OSCC research, while also highlighting the need for stronger *in vivo* and early clinical evidence to better define its therapeutic relevance.

## Data Availability

Not applicable.
